# Aberrantly Expressed Genes and miRNAs in Slow Transit Constipation Based on RNA-Seq Analysis

**DOI:** 10.1155/2018/2617432

**Published:** 2018-08-14

**Authors:** Shipeng Zhao, Qiang Chen, Xianwu Kang, Bin Kong, Zhuo Wang

**Affiliations:** Department of Second Anorectal Section, The Third Hospital of Hebei Medical University, Shijiazhuang 050051, Hebei, China

## Abstract

**Background:**

This study aims to identify the key genes and miRNAs in slow transit constipation (STC).

**Methods:**

MRNA and miRNA expression profiling were obtained. Differentially expressed genes (DEGs) and miRNAs were identified followed by the regulatory network construction. Functional annotation analysis and protein-protein interaction (PPI) network were conducted. The electronic validation was performed.

**Results:**

Hsa-miR-2116-3p, hsa-miR-3622a-5p, hsa-miR-424-5p, and hsa-miR-1273-3p covered most DEGs. HLA-DRB1, HLA-DRB5, C3, and ICAM were significantly involved in staphylococcus aureus infection. The PPI network generated several hub proteins including ZBTB16, FBN1, CCNF, and CDK1. Electronic validation of HLA-DRB1, PTGDR, MKI67, BIRC5, CCNF, and CDK1 was consistent with the RNA-sequencing analysis.

**Conclusion:**

Our study might be helpful in understanding the pathology of STC at the molecular level.

## 1. Introduction

Constipation is a gastrointestinal disorder characterized by infrequent bowel movements, defecation difficulty, and incomplete bowel evacuation sensation [1–3]. Constipation is more widespread in women and is often increased with age [4, 5]. It is reported that constipation is associated with inflammatory bowel disease, irritable bowel syndrome, and relevant comorbidities [6]. Additionally, medications, mobility loss, gut aging alterations, and rectal sensory-motor dysfunction can cause constipation. It is worth mentioning that slow transit constipation (STC) is the prototype of functional constipation and frequently appears in general medicine and clinical practice (such as gastroenterology and geriatrics). Bharucha AE et al. found that STC was related to endocrine and metabolic disorders including hypercalcemia, hypothyroidism, or diabetes mellitus [7].

Recently, the underlying pathophysiology of STC is poor to predict and physiological assessment of gastrointestinal tract transit is the common diagnostic method. Taking laxative remains the primary treatment option in severe STC [3, 8] and patients may take laxative for many years in many instances. Despite advances in therapy of STC, treatment failure is a common phenomenon [9]. Therefore, exploring the underlying pathology mechanism is urgent. In physiological conditions, colon motor activity increases after meals and wake up and decreases during sleep [10]. And STC is characterized by prolonged transit time of the stool through the colon on account of the reduced colonic transit rate [11]. Maybe, altered colonic function plays an important role in patients with STC.

To our knowledge, expression profiling of mRNAs and miRNAs in STC colon tissue through RNA-sequencing has not been reported. In this study, we used high-throughput RNA-sequencing to study the mRNA and miRNA expression profiling and investigated the functional significance of aberrantly expressed genes and miRNAs in STC.

## 2. Methods

### 2.1. Patients and Samples

In this study, we selected patients with slow transit constipation (STC) strictly according to international or domestic standard. Those patients with drug-induced constipation, other diseases (parkinson, type 1 diabetes, rheumatoid arthritis, systemic sclerosis, systemic lupus erythematosus) induced STC, large intestine organic disease, giant colon or colorectal neoplasms, constipation patients with defecation disorder, normal transit, and mixed types were excluded. For normal control group, only those individuals with no defecation disorder, anus and rectum organic disease, history of constipation, or the laxative use were included. Finally, 3 patients (2 women and 1 man) were diagnosed as slow transit constipation (STC) and 3 patients with colon cancer were enrolled into our study. The mean age of 3 STC patients was 56 years (range: 48-61). The left half colon tissue of in STC patients and left half paracarcinoma colon tissue in patients with colon cancer were obtained through surgical resection. The clinical information of patients with STC and control individuals was shown in Table 1.

In addition, our study was approved by the ethics committee of the local hospital and informed written consent was obtained from all patients. And colon tissue of STC patients and paracarcinoma colon tissue of colon cancer patients were obtained based on the Helsinki Declaration.

### 2.2. RNA Isolation and Sequencing

Total RNA was isolated from samples by using the Trizol reagent (Invitrogen, Carlsbad, CA, USA). The Nanodrop ND-2000 spectrophotometer (Thermo Scientific, Wilmington, DE, USA) was used to check the RNA concentration and purity. 1.5% agarose gel electrophoresis was applied to test the integrity of RNA. Then, RIN value (RIN>7) was obtained through an Agilent 2100 Bioanalyzer. The messenger RNA (mRNA) was purified by oligo-d (T) probes for polyA selection. And the purified mRNA was fragmented into size of 200 bp by TruSeq RNA library preparation kit (Illumina, San Diego, CA, USA). The complementary DNA (cDNA) was generated. End repair and adapter ligation were performed after purification by QIAquick PCR purification kit. The gel purification (2% TAE) was performed to isolate 300 nt fragments. The mRNA library was constructed by QIAquick PCR and 18-30 nt RNA was obtained from the total RNA. Adapter ligation and reverse transcription PCR were performed to obtain the cDNA by TruseqTM Small RNA sample prep kit. Finally, sequencing was performed by A HiSeqTM 2500/Miseq platform (Illumina).

### 2.3. Quality Control of Raw Sequence Data

The raw image data of mRNAs and miRNAs derived from high-throughput RNA-sequencing was translated into raw FASTQ sequence data by using Base-Calling. Quality control of FASTQ data (Read QC) was performed by using FastQC v0.11.4. To obtain the high quality clean data, the low quality sequences (adaptor sequences, sequences with a quality score <20, and sequences with N base rate of raw reads >10%) were removed from sequencing data of mRNAs and miRNAs by using cutadapt v1.9.1.

### 2.4. Identification of DEGs in STC

According to human UCSC genome reference annotation, the alignment between cleaned mRNA sequencing reads was aligned to the human genome (GRCh38.p7 assembly) via Tophat (http://tophat.cbcb.umd.edu/). The fragment was performed to assemble and relative expression of the reads with the normalized RNA-sequencing fragment was counted by using Cufflinks (http://cufflinks.cbcb.umd.edu/). Fragments per kilobase of exon per million mapped reads (FPKM) were calculated by Cuffdiff (http://cufflinks.cbcb.umd.edu/) to determine the transcription abundance of mRNAs. Paired t-tests were performed to test the expression between STC and controls. DEGs in STC compared to controls were identified with *p* value < 0.05 and abs (log2 (fold change)) > 1. The heat map of top 100 DEGs in STC was obtained by heatmap.2 (http://www.bioconductor.org/packages/release/bioc/html/heatmaps.html).

### 2.5. Identification of Differentially Expressed miRNAs in STC

Based on human genome UCSC reference annotation, the alignment between cleaned miRNA sequencing reads was aligned to the human genome (GRCh38.p7 assembly) via bowtie (bowtie-bio.sourceforge.net). And miRDeep2 (https://www.mdc-berlin.de/8551903/en/) was applied to determine the transcription abundance of miRNAs. The differentially expressed miRNAs in STC compared to controls were calculated by using DESeq2 (http://bioconductor.org/packages/DEGseq/) and the threshold was defined as *p* value < 0.05 and abs (log2 (fold change)) > 1. The heat map of differentially expressed miRNAs in STC was obtained by heatmap.2 (http://www.bioconductor.org/packages/release/bioc/html/heatmaps.html).

### 2.6. Network Construction of Differentially Expressed miRNA Targets

Identifying target genes is an important step in studying the function of miRNA in tissues. In this study, the DEGs whose expression was inverse with corresponding differentially expressed miRNAs were obtained and checked by six bioinformatic algorithms (RNA22, miRanda, miRDB, miRWalk, PICTAR2, and Targetscan). Those targets recorded by more than 4 algorithms were selected. And verified targets were also obtained by miRWalk (http://www.umm.uni-heidelberg.de/apps/zmf/mirwalk/). According to the differentially expressed miRNA-target pairs, the STC-specific differentially expressed miRNA-targets interaction network was established by Cytoscape software (http://www.cytoscape.org/).

### 2.7. Functional Annotation of Differentially Expressed miRNA Targets

In order to study the biological function of differentially expressed miRNA-targets, the Gene Ontology (GO) and Kyoto Encyclopedia of Genes and Genomes (KEGG) pathway analysis were performed by using the online software GeneCodis (http://genecodis.cnb.csic.es/analysis). And the threshold of FDR<0.05 was set as the criteria of statistical significance.

### 2.8. Protein-Protein Interaction Analysis

In order to understand the protein interaction between DEGs in STC, BioGRID database was utilized to select interacting protein pairs. And the protein-protein network (PPI) was visualized by Cytoscape software (http://cytoscape.org/). In the network, nodes represent proteins and edges represent interaction between two proteins.

### 2.9. Validation the Expression of DEGs and Differentially Expressed miRNAs by GEO

In order to validate the identified DEGs and differentially expressed miRNAs in STC, the Gene Expression Omnibus (GEO, http://www.ncbi.nlm.nih.gov/geo) database was used for electronic validation. We compared expression levels of selected DEGs and hsa-miRNAs between STC cases and normal controls and the difference of expression levels was showed by box-plots.

## 3. Results

### 3.1. DEGs and Differentially Expressed miRNAs in STC

**Figure 1:**
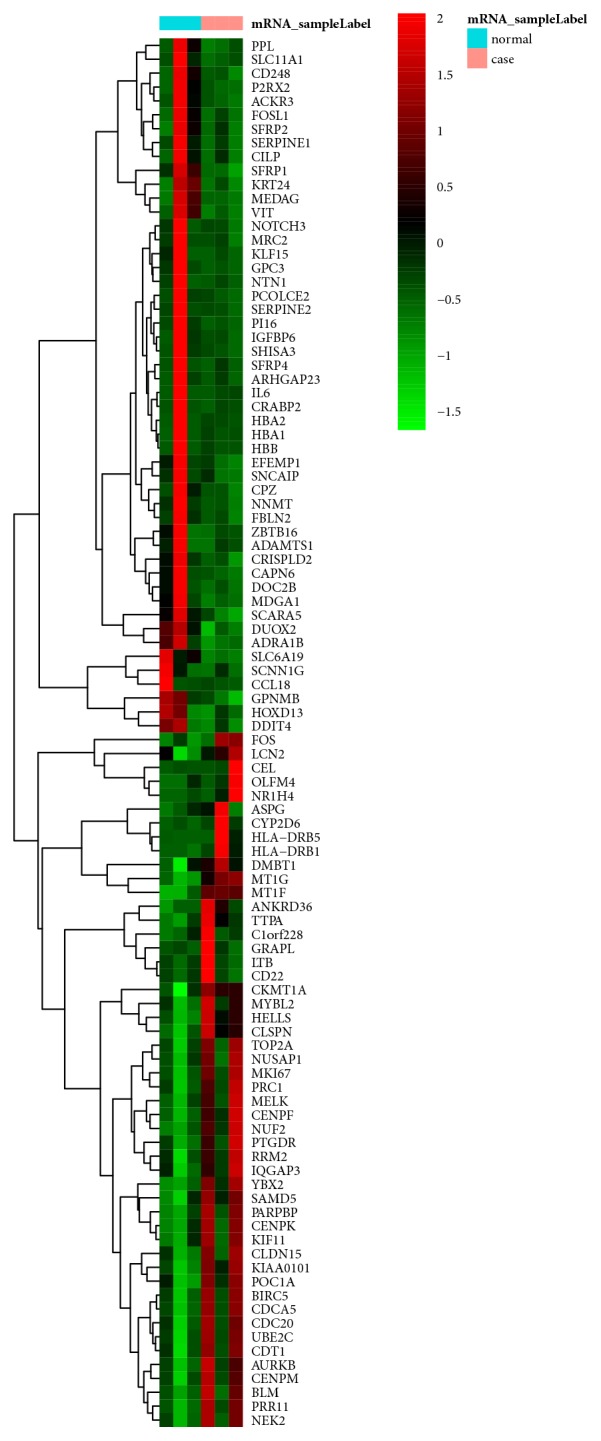
Hierarchical clustering analysis based on the expression profile of the top 100 DEGs in STC compared to normal tissues. Orange and light blue color mean the group of STC and normal tissues, respectively. Red color represents the relative expression level of genes which was higher than mean, and green color represents the relative expression of genes which was lower than mean.

**Figure 2:**
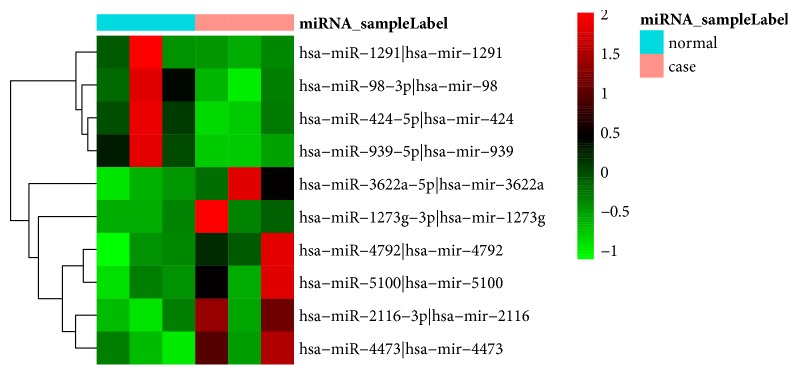
Hierarchical clustering analysis based on the expression profile of all differentially expressed miRNAs in STC compared to normal tissues. Orange and light blue color mean the group of STC and normal tissues, respectively. Red color represents the relative expression level of genes which was higher than mean, and green color represents the relative expression of genes which was lower than mean.

A total of 464 DEGs (357 upregulated and 107 upregulated genes) and 10 differentially expressed miRNAs (4 upregulated and 6 downregulated miRNAs) were obtained. Heap maps of top 100 DEGs and all differentially expressed miRNAs were displayed in Figures 1 and 2, respectively. The top 20 DEGs were shown in Table 2.

### 3.2. Differentially Expressed miRNAs-Targets Network

**Figure 3:**
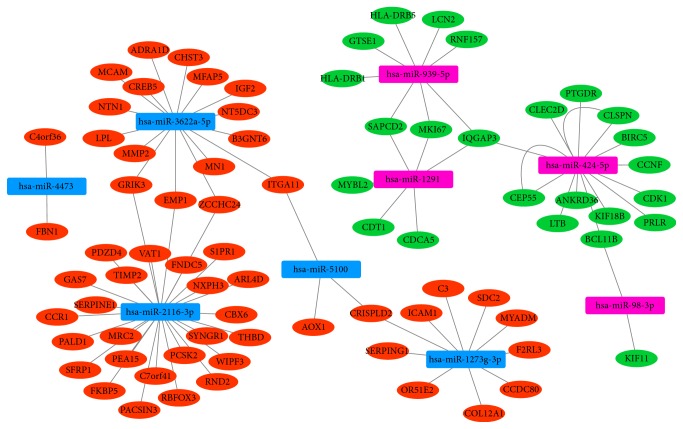
STC-specific differentially expressed miRNA-targets interaction network. Ellipses were used to represent the DEGs and red and green color were used to represent up- and downregulation in STC, respectively. Rectangles were used to represent differentially expressed miRNAs and rose and blue color were used to represent up- and downregulation, respectively.

A total of 87 differentially expressed miRNAs-target pairs including 25 upregulated miRNA-downregulated target pairs and 47 downregulated miRNA-upregulated target pairs were obtained. Among which, there were 17 differentially expressed miRNAs-target pairs that have been confirmed by miRWalk. Based on the STC-specific differentially expressed miRNAs-target interaction network (Figure 3), hsa-miR-2116-3p (degree=27), hsa-miR-3622a-5p (degree=16), hsa-miR-424-5p (degree=15), and hsa-miR-1273-3p (degree=10) were the top four differentially expressed miRNAs covered most DEGs.

### 3.3. Functional Annotation of Differentially Expressed miRNAs Targets

**Figure 4:**
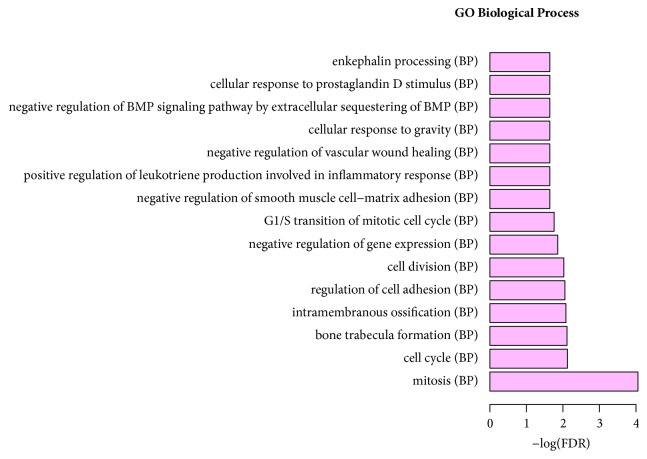
Significantly enriched biological function of target DEGs in STC.

**Figure 5:**
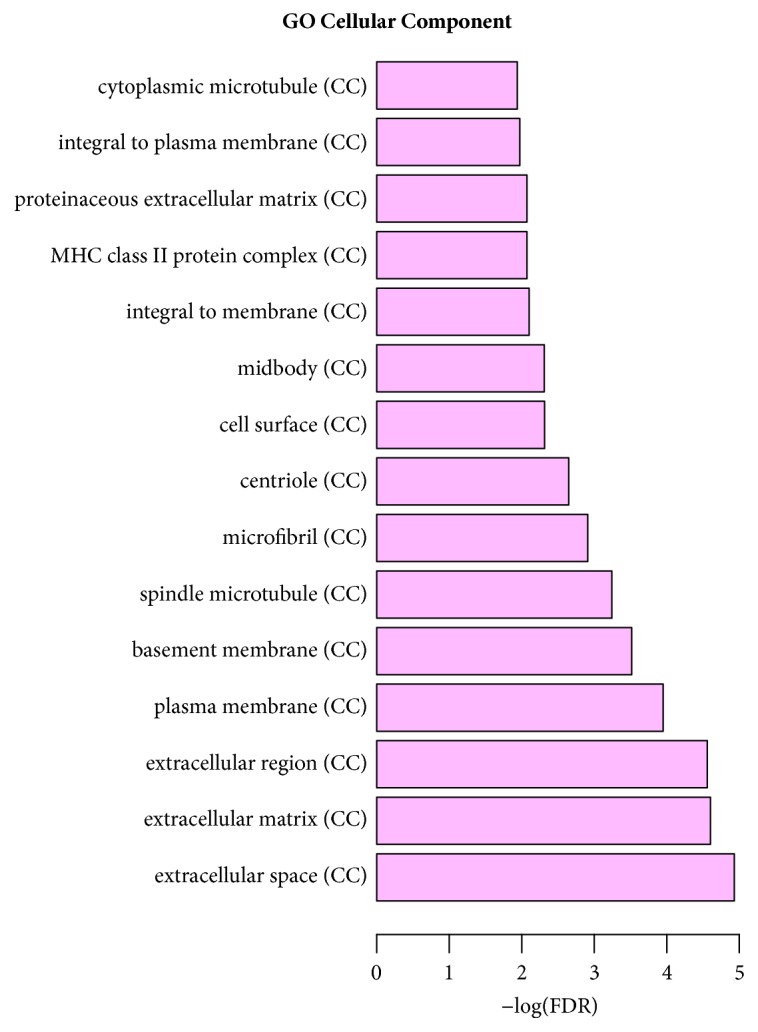
Significantly enriched cellular component of target DEGs in STC.

**Figure 6:**
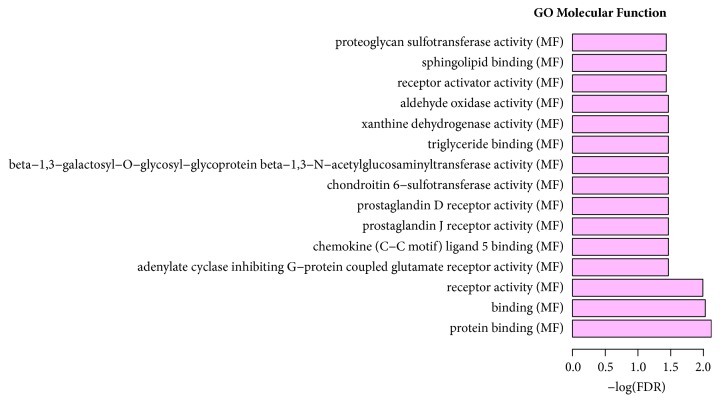
Significantly enriched molecule function of target DEGs in STC.

**Figure 7:**
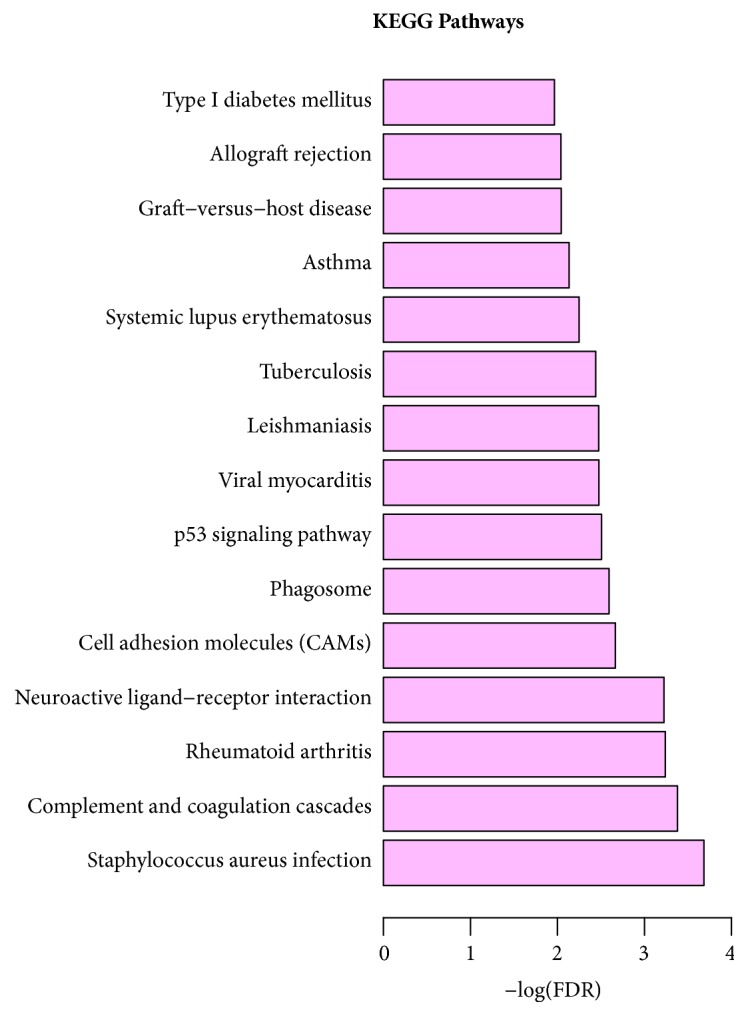
Significantly enriched KEGG pathways of target DEGs in STC.

According to the GO enrichment analysis, mitosis (FDR=0.0000884705), regulation of cell adhesion (FDR=0.00882985), negative regulation of smooth muscle cell-matrix adhesion (FDR=0.0228017), extracellular space (FDR=0.0000117125), centriole (FDR=0.00225184), extracellular region (FDR=0.0000276714), receptor activity (FDR=0.0343115), protein binding (FDR=0.00758689), and binding (FDR=0.00940741) were the most significantly enriched GO terms in STC. The enriched GO terms of target DEGs were shown in Figures 4, 5, and 6, respectively. Cell adhesion molecules (FDR=0.00216279) involving four DEGs (SDC2, HLA-DRB5, ICAM1, and HLA-DRB1), staphylococcus aureus infection (FDR=0.000207533) involving four DEGs (C3, HLA-DRB5, ICAM1, and HLA-DRB1), and neuroactive ligand-receptor interaction (FDR=0.00059726) involving six DEGs (F2RL3, S1PR1, PRLR, GRIK3, ADRA1D, and PTGDR) were three significantly enriched pathways in STC (Figure 7).

### 3.4. Protein-Protein Interaction Network

**Figure 8:**
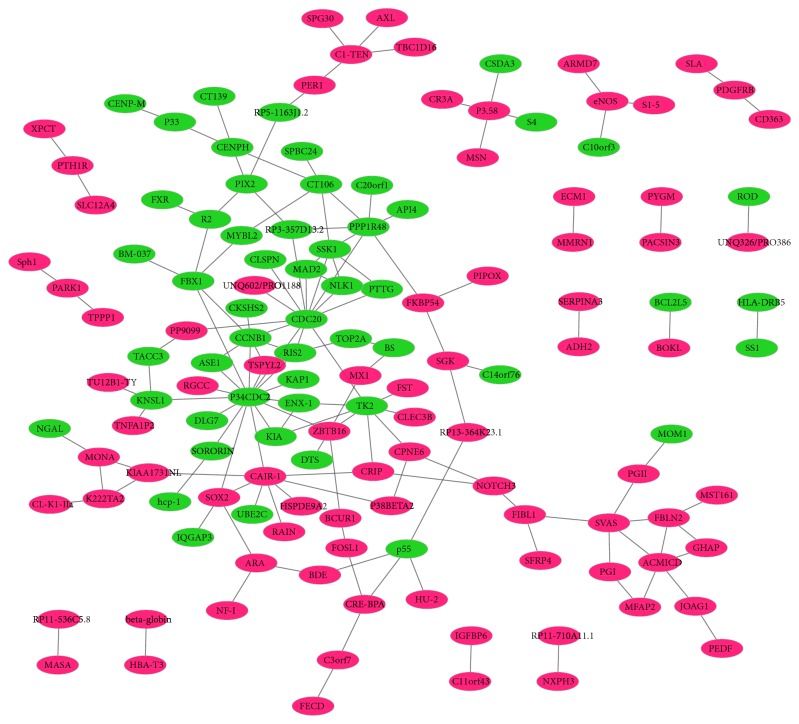
The protein-protein interaction networks of all DEGs in STC. Red and green rectangle nodes represent up- and downregulated DEGs, respectively. The solid line means the interaction correlation between proteins.

**Figure 9:**
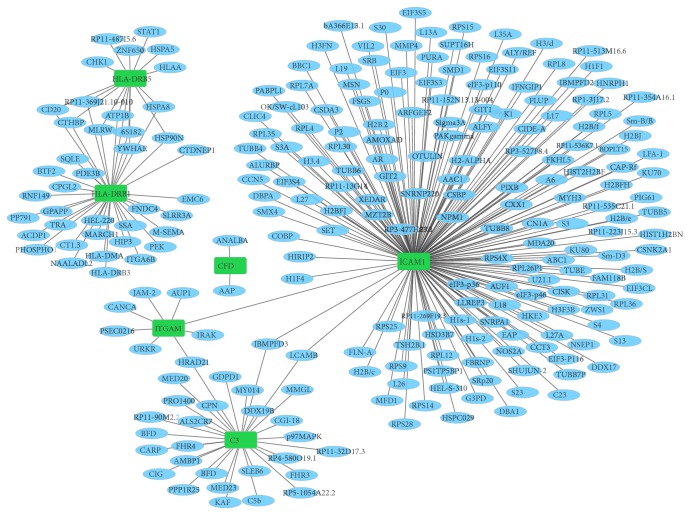
The protein-protein interaction networks of DEGs involved in the staphylococcus aureus infection signaling pathway. Green rectangle nodes represent DEGs. The solid line means the interaction correlation between proteins.

PPI network of all DEGs in STC was determined by Cytoscape software. The proteins which had the high connectivity with other proteins were hub proteins. In the PPI network, CDK1 (degree=16), CDC20 (degree=13), TK1 (degree=8), BAG3 (degree=8), CCNB1 (degree=7), AURKB (degree=7), BUB1B (degree=6), FBN1 (degree=5), ELN (degree=5), CCNF (degree=5), ZBTB16 (degree=5), and NUF2 (degree=5) were the hub proteins (Figure 8). Among which, we also performed the PPI network of several DEGS including C3, HLA-DRB5, ICAM1 and HLA-DRB1, ITGAM, and CFD that involved in the staphylococcus aureus infection signaling pathway (Figure 9).

### 3.5. Validation the Expression of DEGs and Differentially Expressed miRNAs by GEO

**Figure 10:**
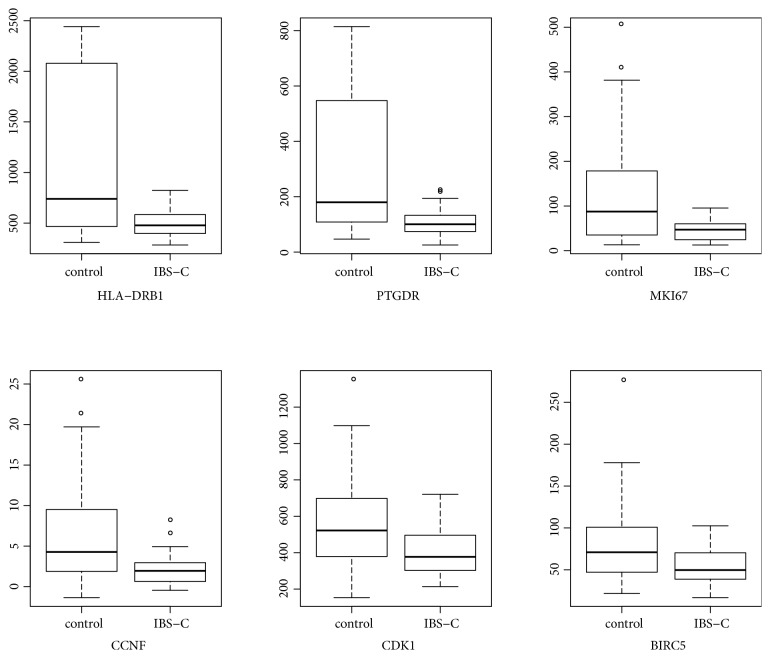
The electronic validation box-plots of DEGs and differentially expressed miRNA in the GEO dataset.

Based on the RNA-sequencing data, six downregulated DEGs (HLA-DRB1, PTGDR, MKI67, BIRC5, CCNF, and CDK1) in STC were selected to perform the expression validation by GEO dataset (Figure 10). Different expression levels of these genes and miRNAs were analyzed and depicted through box-plots. HLA-DRB1, PTGDR, MKI67, BIRC5, CCNF, and CDK1 were significantly downregulated in the case group compared to the normal control group, which was consistent with our RNA-sequencing data.

## 4. Discussion

STC is an intestine disease that influences the health and life quality of patients. Therefore, understanding the pathology mechanism will make a contribution to reducing the incidence rate of STC. In our study, we identified several DEGs and differentially expressed miRNAs in STC, which may play roles in the molecular level of STC.

In top 20 DEGs, we found two cell proliferation-related genes including MKI67 and BIRC5 in colon tissue of STC. Marker of proliferation Ki-67 (MKI67) is a cellular proliferation marker that is detectable in the cell nucleus during active phases of the cell cycle except resting cells [12]. It has been demonstrated that MKI67 plays an important role in colon cancer [13]. Herein, we also found that MKI67 was one of target genes of hsa-miR-1291 in STC. It has been indicated that hsa-miR-1291 can promote apoptosis in esophageal squamous cell carcinoma by regulating mucin1 [14]. Additionally, it is reported that hsa-miR-1291 is upregulated in colon cancer [15]. Baculoviral IAP repeat containing 5 (BIRC5, also called survivin) is a proliferation marker and belongs to the members of the IAP family of proteins [16, 17]. BIRC5 controls a number of cellular processes including cell apoptosis [18]. It is found that BIRC5 is highly expressed in colon cancer cell lines [19]. And increased expression of BIRC5 is related to poor clinical outcome in patients with colon cancer patients [20]. In addition, it is differentially expressed in irritable bowel syndrome rectosigmoid mucosa [21]. In our study, we found downregulated expression of cell proliferation markers of BIRC5 and MKI67 in the colon tissue of STC, which indicated that BIRC5 and MKI67 may be involved in the cell proliferation and apoptosis in the development of STC.

Microbiota infection is common in different intestine disease. Postinfectious irritable bowel syndrome (PI-IBS) presents a bout of gastroenteritis caused by viral, parasitic, or bacterial infections [22]. It is worth mentioning that staphylococcus aureus infection is a potential pathogenic factor in PI-IBS [23–26]. It is reported that inflammatory bowel disease causes inflammatory obstruction of the gastrointestinal tract, resulting in symptoms such as constipation [27]. This suggested the correlation between inflammation and constipation. In this study, we found that staphylococcus aureus infection was the most significantly enriched pathway of DEGs in STC. And four DEGs including HLA-DRB1, HLA-DRB5, C3, and ICAM1 were involved in this signal pathway.

Major histocompatibility complex, class II, DR beta 1 (HLA-DRB1) has been demonstrated to be associated with ulcerative colitis and Crohn's disease [28, 29]. Furthermore, HLA-DRB1 is differentially expressed in precancerous colorectal adenomas mucosa [30]. Another major histocompatibility complex family member, major histocompatibility complex, class II, DR beta 5 (HLA-DRB5) is found significantly upregulated in human colorectal cancer epithelial cell and has prognostic value in the prediction of colon cancer metastasis [31, 32]. Herein, we found downregulated expression of both HLA-DRB1 and HLA-DRB5 in STC. Interestingly, they were under the regulation of hsa-miR-939-5p. It is reported that low expression of hsa-miR-939 is significantly associated with shorter distant metastasis-free survival of colon cancer and has a prognostic value in the development of colon cancer [33]. It is demonstrated that complement C3 (C3) is locally synthesized and secreted in the human intestine [34]. It is first found that the C3 mRNA expression is significantly upregulated in the inflamed mucosa of patients with inflammatory bowel disease [35]. It is worth mentioning that the expression of C3 is downregulated in the distal colon of the rat with constipation and decreased after the laxative treatment [36]. Intercellular adhesion molecule 1 (ICAM1) is found to be involved in the colon cancer cell proliferation [37]. In this study, we found upregulated expression of both C3 and ICAM in STC. In addition, the PPI network of HLA-DRB1, HLA-DRB5, C3, and ICAM1 showed that these genes had a significant association with proteins encoded by other differentially expressed genes. Our result suggested that HLA-DRB1, HLA-DRB5, C3, and ICAM1 may play a proinflammatory role in the development of STC.

In addition, we found that C3 and ICAM were commonly regulated by hsa-miR-1273 g-3p in STC. Hsa-miR-1273 has been regarded as a functionally uncharacteristic miRNAs in colon cancer exosomes [38]. In addition, another two upregulated DEGs including F2R like thrombin or trypsin receptor 3 (F2RL3) and syndecan 2 (SDC2) were also target genes of hsa-miR-1273 g-3p in STC. F2RL3 (also called PAR4) is a protease that plays roles in antinociceptive effects in visceral sensitivity through protease-activated receptor [39]. The expression of F2RL3 is downregulated in the colon of patients with irritable bowel syndrome patients [40, 41], while, it is significantly upregulated in patients with colon cancer and ulcerative colitis [42, 43]. SDC2 functions as a cell surface receptor and plays roles in interacting with extracellular matrix components and cell-to-cell signaling molecules [44]. It is found that SDC2 is low methylated in peripheral blood DNA of colon cancer and serves as a blood-based diagnostic marker of colon cancer [45]. In a word, our result suggested that the expression of C3, ICAM1, F2RL3, and SDC2 under the regulation of hsa-miR-1273 g-3p may be important molecules in the process of STC.

In the PPI network, we found several high degree proteins encoded by DEGs, such as zinc finger and BTB domain containing 16 (ZBTB16), fibrillin 1 (FBN1), cyclin F (CCNF), and cyclin dependent kinase 1 (CDK1). Promyelocytic leukemia zinc finger protein (PLZF), encoded by* ZBTB16* gene, is involved in different growth regulatory and differentiation pathways. It is significantly upregulated from normal colonic mucosa to aberrant crypt foci and colon cancer [46]. FBN1 is an extracellular matrix glycoprotein and high methylation in colonic tissue of patients with colon cancer [45]. The genetic mutation of FBN1 is also associated with colon cancer [47]. It is noted that FBN1 has been identified as a promising early detection biomarker of colon cancer and colon adenomas [48]. In this study, the expressions of ZBTB16 and FBN1 were upregulated and under the regulation of hsa-miR-4473 in STC. Our result suggested that both ZBTB16 and FBN1 could be involved in the growth regulatory of STC.

CCNF contributes to the G2 to M phase transition in the cell cycle. It is a key gene in colorectal adenoma-to-cancer progression [49]. It is reported that CCNF is expressed in colon cancer tissue samples and overexpressed as an epigenetic clock gene in colon adenoma tissue samples [50, 51]. It is reported that the interaction between CDK1/cyclin B1 complex and ZNFX1 antisense RNA 1 (ZFAS1) leads to cell cycle progression and cell apoptosis suppression in the progression of colon cancer [52]. It is found that CDK1 is remarkably downregulated in colon cancer cell lines [53]. Significantly, it is regarded as a promising prognostic biomarker of the metastasis risk in stage II colon cancer [54]. In this study, CCNF and CDK1 were downregulated and regulated by hsa-miR-424-5p in STC. It is found that hsa-miR-424 is significantly upregulated in colon cancer [55]. In addition, hsa-miR-424 is a promising biomarker for the early diagnosis of colon cancer [56]. Beside CCNF and CDK1, prostaglandin D2 receptor (PTGDR) is also the target gene of hsa-miR-424 in STC. It is found that the expression of PTGDR is lower in colon cancer [57]. It is proposed that hypermethylation of PTGDR may make a contribution to reducing gene expression and developing colon cancer [58]. In a word, our study suggested that CCNF, CDK1, and PTGDR may be involved in the cell cycle progression of STC.

It is reported that hsa-miR-2116 presents remarkable differential expression when there are responses to bacterial infection in human host cells [59]. Herein, we found downregulated expression of hsa-miR-2116-3p in the colon tissue of STC. Furthermore, sphingosine-1-phosphate receptor 1 (S1PR1) and serpin family E member 1 (SERPINE1) were also target genes of hsa-miR-2116-3p. In mice, genetic deletion of* S1pr1 *will increase vascular permeability in the colon and enhance bleeding in colitis [60]. Additionally, it is found that targeting SphK1/S1P/S1PR1 may be a potential therapeutic option to prevent the development from colitis to colon cancer [61]. SERPINE1 is an inhibitor of plasmin action and has been indicated to promote cancer invasion [62]. It is found that SERPINE1 is upregulated in colon cancer and has been considered as a biomarker associated with poor prognosis in colon cancer [63]. Hamfjord et al. first found the expression of hsa-miR-3622a-5p in colon cancer tissues [64]. In this study, we found downregulated expression of hsa-miR-3622a-5p in colon tissue of STC. And adrenoceptor alpha (ADRA1D) was one of target genes of hsa-miR-3622a-5p in STC. It is found that ADRA1D is differentially expressed in stage IV compared to stages II and III colon cancer [65]. It is reported that hsa-miR-98 is upregulated in active ulcerative colitis [66]. Furthermore, the upregulation of hsa-miR-98 could be considered as the molecular marker for the colon in active ulcerative colitis patients [66]. Our result indicated that these hsa-miRNAs and target genes may play an important role in the development of STC.

Beside the abovementioned hsa-miRNAs, hsa-miR-5100 and hsa-miR-4792 were also found in the colon tissue of STC. However, there were no previous reports about the relationship between hsa-miR-5100, hsa-miR-4792, and intestine disease including STC. Therefore, further research is needed to study the function of hsa-miR-5100 and hsa-miR-4792 in STC.

## 5. Conclusion

In summary, we identified 464 DEGs and 10 differentially expressed miRNAs in STC compared to normal tissues. PPI network generated some hub proteins such as ZBTB16, FBN1, CCNF, and CDK1. Target DEGs of differentially expressed miRNAs were significantly enriched in staphylococcus aureus infection involving four DEGs (HLA-DRB1, HLA-DRB5, C3, and ICAM), which suggested that inflammation may be a factor for STC. Our study was the first article for exploration of gene expression profiling in colon tissue of STC. Our findings may provide the fundamental work for understanding the molecular pathology of STC. However, there are limitations to our study. Firstly, the sample size in the RNA-sequencing was small and large numbers of samples of STC are needed for further research. Secondly, these dysregulated genes, miRNAs, and related signaling pathways in STC were identified and the potential molecular mechanism was not studied. Therefore, in vivo and in vitro experiments including animal model and cell culture were necessary to elucidate the underlying pathological mechanism of STC in the future work. Thirdly, qRT-PCR is needed to further validate the expression of several differentially expressed mRNAs and miRNAs.

## Figures and Tables

**Table 1:** The basic characteristic of patients with STC and control individuals.

**Patient/Control**	**Age**	**Gender**	**Time (years)**	**Histological feature**	**Preoperative defecate frequency** **(time/day)**
1 (Patient)	59	Female	30	STC	1 /4
2 (Patient)	48	Female	30	STC	1/5-15
3 (Patient)	61	Male	1	STC	1/4
4 (Control)	69	Male	N/A	Normal	3-4/1
5 (Control)	47	Male	N/A	Normal	5-6/1
6 (Control)	64	Female	N/A	Normal	6-7/1

**Table 2:** Top 20 DEGs in STC.

**Gene**	**log2.fold_change.**	**test_stat**	**p_value**	**Up_down**
CD248	1.90069	2.44353	5.00E-05	up
GPC3	2.85031	2.85269	5.00E-05	up
HBA2	2.94208	2.90606	5.00E-05	up
MEDAG	2.96147	2.76909	5.00E-05	up
NNMT	1.94212	2.35541	5.00E-05	up
P2RX2	3.80911	1.88888	5.00E-05	up
PCOLCE2	2.54104	2.50303	5.00E-05	up
PI16	3.85702	3.77055	5.00E-05	up
SLC6A19	2.55631	3.30198	5.00E-05	up
ZBTB16	1.99184	2.33881	5.00E-05	up
HLA-DRB5	-5.37709	-4.12938	5.00E-05	down
YBX2	-2.14855	-2.67695	5.00E-05	down
OLFM4	-2.49538	-2.20077	0.0001	down
HLA-DRB1	-2.0477	-2.40945	0.0003	down
FOS	-1.63902	-1.96379	0.0005	down
MKI67	-1.70928	-2.14918	0.00075	down
MT1G	-1.36954	-1.9624	0.00075	down
HELLS	-1.84715	-1.66191	0.00085	down
PTGDR	-1.73283	-1.82581	0.0013	down
BIRC5	-1.59705	-1.83121	0.0015	down

## Data Availability

The data used to support the findings of this study are included within the article.
